# Organic Thin Films Based on DPP-DTT:C60 Blends Deposited by MAPLE

**DOI:** 10.3390/nano10122366

**Published:** 2020-11-27

**Authors:** Marcela Socol, Nicoleta Preda, Carmen Breazu, Andreea Costas, Gabriela Petre, Anca Stanculescu, Gianina Popescu-Pelin, Andreea Mihailescu, Gabriel Socol

**Affiliations:** 1 National Institute of Material Physics, 405A Atomistilor Street, 077125 Magurele, Romania; carmen.breazu@infim.ro (C.B.); andreea.costas@infim.ro (A.C.); gabriela.petre@infim.ro (G.P.); sanca@infim.ro (A.S.); 2 Faculty of Physics, University of Bucharest, 405 Atomistilor Street, 077125 Magurele, Romania; 3 National Institute for Lasers, Plasma and Radiation Physics, 409 Atomistilor Street, 077125 Magurele, Romania; gianina.popescu@inflpr.ro (G.P.-P.); andreea.mihailescu@inflpr.ro (A.M.); gabriel.socol@inflpr.ro (G.S.)

**Keywords:** MAPLE, organic thin films, conjugated polymer DPP-DTT, fullerene C60, photovoltaic cell

## Abstract

The matrix-assisted pulsed laser evaporation (MAPLE) technique was used for depositing thin films based on a recently developed conjugated polymer, poly[2,5-(2-octyldodecyl)-3,6-diketopyrrolopyrrole-alt-5,5-(2,5-di(thien-2-yl)thieno [3,2-b]thiophene)] (DPP-DTT) and fullerene C60 blends. The targets used in the MAPLE process were obtained by freezing chloroform solutions with different DPP-DTT:C60 weight ratios, with the MAPLE deposition being carried at a low laser fluence, varying the number of laser pulses. The structural, morphological, optical, and electrical properties of the DPP-DTT:C60 blend layers deposited by MAPLE were investigated in order to emphasize the influence of the DPP-DTT:C60 weight ratio and the number of laser pulses on these features. The preservation of the chemical structure of both DPP-DTT and C60 during the MAPLE deposition process is confirmed by the presence of their vibrational fingerprints in the FTIR spectra of the organic thin films. The UV-VIS and photoluminescence spectra of the obtained organic layers reveal the absorption bands attributed to DPP-DTT and the emission bands associated with C60, respectively. The morphology of the DPP-DTT:C60 blend films consists of aggregates and fibril-like structures. Regardless the DPP-DTT:C60 weight ratio and the number of laser pulses used during the MAPLE process, the current–voltage characteristics recorded, under illumination, of all structures developed on the MAPLE deposited layers evidenced a photovoltaic cell behavior. The results proved that the MAPLE emerges as a viable technique for depositing thin films based on conjugated polymers featured by a complex structure that can be further used to develop devices for applications in the solar cell area.

## 1. Introduction

Among all available renewable energy technologies, photovoltaic technology is the most promising due the notable progress achieved in the last years [1,2,3]. Thus, over the past two decades the development of flexible, lightweight, and low-cost organic photovoltaic devices based on polymers has captured the interest of the scientific community, becoming one a hot research topic [4,5,6]. Special attention was paid to find and understand the link between the molecular design of the conjugated polymers, their morphology and optical properties, and the electrical performance of the polymer solar cell (PSC) architecture develop on such organic compounds featured by semiconducting properties. A study published in 2020 reports the fabrication of a PSC which achieved an over 18% efficiency, this being the highest value reported in the field of organic solar cells [7]. The increasing interest in photovoltaic technology based on semiconducting polymers is motivated by the advantages of the PSCs over their inorganic counterparts, including their compatibility with plastic (flexible) substrate, transparency, large area coverage, low manufacturing costs due to the roll-to-roll production, being easy to integrate into other products, low environmental impact, and short energy payback times [8,9]. Therefore, PSCs can be regarded as key building blocks for future organic photovoltaic devices.

Poly(3-hexylthiophene) (P3HT) has been widely used in PSCs because this semiconducting polymer is characterized by a band gap of ~1.9 eV, with it being known that the value of this parameter influences the device performance due to the better light harvesting [4]. Although an efficiency of about 5% was achieved using P3HT, the HOMO energy position at −5.0 eV of this polymer [10] is still a major limitation. For this reason, many efforts have been made to obtain narrow band gap polymers by alternating electron-rich units (donor) and electron-deficient units (acceptor) covalently bonded within the same chain, it being known that the weak donors maintain a low HOMO energy level and the strong acceptors reduce the bandgap by promoting intramolecular charge transfer (ICT) from donor to acceptor moieties [4,10,11]. Thus, an efficiency of over 10% was reported for a cell structure containing a blend of a semiconducting polymer with a band gap of 1.59 eV (poly[2,6′-4,8-di(5-ethylhexylthienyl)benzo[1,2-b;3,3-b]dithiophene] [3-fluoro-2[(2-ethylhexyl)carbonyl]thieno [3,4-b]thiophenediyl] (PTB7-Th) and a fullerene derivative compound [6,6]-phenyl C71-butyric acid methyl ester (PC71BM) [12].

Recently, poly[2,5-(2-octyldodecyl)-3,6-diketopyrrolopyrrole-alt-5,5-(2,5-di(thien-2-yl)thieno[3,2-b]thiophene)] (DPP-DTT) has emerged as an excellent narrow band gap polymer (band gap of 1.7 eV, with a HOMO level of −5.2 eV and a LUMO level of −3.5 eV) which was successfully applied in the PSC and organic field-effect transistor (OFET) fields due to its high hole mobility (10 cm^2^/Vs) and stability [5,13,14,15]. An important advantage presented by solar cells involving DPP-DTT consists of the fact that the photovoltaic response is obtained even for films with a thickness greater than 300 nm, it being known that the thickness of the active layer in the cells based on organic materials is limited owed to the short diffusion lengths of the excitons [4,14,16]. Hence, organic films with a small thickness feature weak absorption, this being responsible for the low number of the excitons generated in the active layer [4]. Accordingly, a compromise between the thickness and the light harvesting must be reached.

Fullerene C60 is another organic compound characterized by a high mobility that is frequently used in organic photovoltaic cells (OPV), either as a thin film in the OPV with stacked layers or as blends in mixture with other materials in the OPV with bulk heterojunction (BHJ) [17,18,19]. Generally, in the solar cells based on conjugated polymer and fullerene blends, the conjugated polymer acts as a p-type material while the fullerene acts as a n-type material [20].

Indisputably, solution-processing techniques have been extremely useful in the development of polymer solar cells [21]. These methods involve relatively low processing temperatures for preserving the chemical structure of the polymer. However, when such solution-based techniques are used for fabricating structures with stacked layers, the different solvents must be adequate in order to avoid damage to the previously deposited layer. This hindrance can be overcome using the active layer as BHJ, with this approach having the advantage of forming a larger donor–acceptor interface which increases the probability of excitons dissociation [22]. From the solution-processing techniques, spin-coating is indubitably the most used in the deposition of organic films involved in the fabrication of solar cells [4,23,24]. To date, to our knowledge spin-coating was the main technique involved in the deposition of thin films based on DPP-DTT and its blends with PC71BM, a fullerene derivative compound [14,25].

Derived from conventional pulsed laser deposition (PLD), matrix-assisted pulsed laser evaporation (MAPLE) is a technique that was developed to process soft organic compounds such as small molecules, oligomers, polymers, or biomaterials without damaging their chemical structure during their deposition [26,27,28]. Compared to the PLD method, in the MAPLE technique the direct interaction of the laser beam with the organic phase is reduced by the use of a solvent which mainly absorbs the incident laser radiation. Thus, in MAPLE deposition solid frozen targets prepared from a solution containing low amounts of raw organic compound (typically below 5 wt.%) dissolved in an appropriate solvent were used. In order to preserve the composition of the evaporated species, the laser fluence values are lower (typically under 500 mJ/cm^2^) than the ablation threshold. After laser irradiation, the solvent and organic molecules are ejected simultaneously from the target. Further, the solvent is pumped away by the vacuum system, while the organic molecules nucleates in the form of a thin film on the substrate’s surface. This approach allows the deposition of organic thin films with the preservation of the chemical structure of the raw material due to the fact that the energy of the laser pulse is absorbed selectively by the solvent, with various theoretical and experimental studies being focused on the mechanism involved in the laser ablation of the organic compounds by MAPLE [29,30,31,32,33]. Deposition parameters such as laser fluence, the number of laser pulses, or the substrate–target distance can be adapted for obtaining organic layers characterized by adequate optical and electrical properties for developing OPV in both configurations, with stacked p-n organic films or with the active layer as bulk heterojunction, the deposition being made even on polyethylene terephthalate (PET) substrate [26,27,34,35,36]. For example, a report reveals that the temperature of the substrate used during the MAPLE process influences the electrical properties of the alkyl-substituted P3HT thin films deposited by this technique [37]. Additionally, a recent study emphasized the influence of the solvent type used in the MAPLE process on the efficiency parameter of the cells based on MAPLE-prepared layers [36].

In the case of thin films based on fullerene and fullerene derivatives deposited by MAPLE, usually these were used as active layers with different organic compounds, in their preparation being involved toluene, chloroform, or dichlorobenzene as solvents [18,26,27,38]. Despite of the C60 low solubility in chloroform, this solvent can be used in MAPLE deposition when the concentration of fullerene is lower than 0.28 g/L, the maximum solubility of C60 in chloroform at 298 K [39]. Regarding the influence of the C60 low solubility on the morphology of the MAPLE deposited layers, it must be mentioned that this parameter cannot play a major role in the agglomeration tendency of C60 molecules during the deposition process, taking into account that such effect was observed for thin films based on azomethine oligomers and C60 deposited by MAPLE using dichlorobenzene as a solvent (known as a good solvent for fullerene).

In this context, the present work is focused on the deposition of thin films based on DPP-DTT:C60 blends by MAPLE in order to emphasize the potential of this “soft” laser-assisted deposition technique for preparing organic thin films with tailored properties for developing organic photovoltaic devices. Chloroform was used as a solvent in the MAPLE deposition based on the following aspects: i) it is a good solvent for DPP-DTT, with this polymer being soluble only in chloroform, chlorobenzene, and dichlorobenzene [14,25], and ii) it features a good absorption in the UV domain [40]. Thus, samples with different DPP-DTT:C60 weight ratios were prepared by varying the number of laser pulses used during the MAPLE process in order to evidence the influence of these two experimental parameters on the structural, morphological, and optical properties of the DPP-DTT:C60 thin films and on the electrical performance of the structures developed on these MAPLE-deposited conjugated polymer-fullerene blend layers.

## 2. Experiment

Conjugated polymer poly[2,5-(2-octyldodecyl)-3,6-diketopyrrolopyrrole-alt-5,5-(2,5-di(thien-2-yl)thieno[3,2-b]thiophene)] (DPP-DTT, Mw ≥ 30,000, PDI ≤ 3) and fullerene C60 were purchased from Ossila (Sheffield, England) while chloroform was acquired from Sigma-Aldrich (Saint Louis, MO, USA). The poly(3,4-ethylenedioxythiophene) polystyrene sulfonate (PEDOT:PSS) solution and ITO/glass with a 20 Ω/square resistance and a roughness lower than 1.8 nm used as substrates for the fabrication of the photovoltaic cells were also bought from Ossila. Thus, before MAPLE deposition, ITO was covered with a PEDOT:PSS thin film using a Chemat Technology spin coater at 6000 rpm speed for 30 s, the obtained coating being annealed for 5 min at 120 °C for improving its stability. Further, this substrate is labelled as ITO/PEDOT:PSS.

In the MAPLE deposition, both organic powders, DPP-DTT and C60, were separately dissolved in chloroform, stirred for several minutes, and then mixed. Further, these solutions with a concentration of 3% weight/volume (*w/v*) containing DPP-DTT:C60 in different weight ratios (1:1, 1:2 and 1:3) were immersed in liquid nitrogen for 30 min to obtain MAPLE solid targets. During the laser process deposition, an excimer laser beam (ArF*, CompexPro 205, Lambda Physics Coherent, Göttingen, Germany) with λ = 193 nm and τ _FWHM_∼ 25 ns was focused on the frozen target [26,27,28]. Hence, when the laser source is focused on the target surface, the laser energy is mainly absorbed by the matrix (solvent), which is vaporized together with the organic molecules. Then, the solvent molecules are pumped away from the deposition chamber, with only the organic molecules reaching on the surface of the substrate, resulting in the formation of the thin film [26]. Throughout the deposition process, the target is constantly rotated with a 0.4 Hz frequency for preventing its local drilling and to improve the deposition uniformity. For all depositions, the target–substrate distance (5 cm) and the laser fluence (100 mJ/cm^2^) were maintained constant. In the same deposition cycle, besides the ITO/glass covered with PEDOT:PSS film used as substrates for developing the photovoltaic structures, glass and silicon were also coated with organic films for the structural, morphological, and optical investigations. Additionally, for comparison reasons, films containing raw organic compounds (DPP-DTT and C60) were deposited by drop casting from the solutions used in the preparation of the MAPLE target films.

The thickness of the organic layers deposited on glass substrates was estimated using an Ambios Technology XP 100 profilometer and given as an average value between three measurements made in different points on each sample.

The vibrational and optical properties of the MAPLE-deposited thin films were evaluated by Fourier transformed infrared spectroscopy (FTIR), UV-VIS spectroscopy, and photoluminescence (PL) techniques. The FTIR spectra were acquired in the 500–3200 cm^−1^ range on a IRTracer-100 (Shimadzu, Kyoto, Japan) spectrometer, the UV-VIS spectra in the 250–1100 nm range on a Thermo Scientific Evolution 220 Spectrophotometer (Waltham, MA, USA) and the PL spectra in the 450–850 nm range at λ_ex_ = 435 nm on an FL920 Edinburgh Instruments Spectrometer (Livingston, UK) with a 435 W Xe lamp excitation and double monochromators on both excitation and emission.

The morphological properties of the MAPLE-deposited thin films were investigated by field emission scanning electron microscopy (FESEM) using a Zeiss Merlin Compact field emission microscope (Oberkochen, Germany) and atomic force microscopy (AFM) involving a Nanonics Multiview 4000 system (Jerusalem, Israel). Additionally, optical images of the organic layers were acquired using the same system implied in the AFM measurements.

For the electrical measurements, lithium fluoride (LiF) and aluminium (Al) films with thicknesses of ~1.5 and ~100 nm, respectively, were deposited as back electrodes on the structures formed on ITO by vacuum evaporation (1.6 × 10^−6^ mbar pressure, substrate at room temperature, substrate rotating) using a Tecuum AG, VCM600-V3-80 (Winterthur, Switzerland) set-up. The current–voltage (J-V) characteristics of the photovoltaic structures developed on the MAPLE-deposited organic films were recorded at room temperature under AM 1.5 illumination using a LOT-Oriel solar simulator (Quantum Design Europe, Darmstadt. Germany) coupled at a Keithley 2602B System Source Meter (Cleveland, OH, USA).

## 3. Results and Discussions

**Figure 1:**
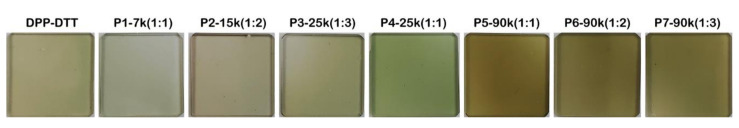
Photographs of thin films based on the DPP-DTT:C60 blends deposited by MAPLE on glass substrate.

In Figure 1, the photographs of the investigated samples reveal that the surface of the substrates was uniformly and completely coated by the organic thin films deposited by MAPLE. The label of the samples based on DPP-DTT:C60 blends (P1–P7) contains the number of laser pulses used during the MAPLE deposition (7 k, 25 k or 90 k) and the DPP:C60 weight ratio (1:1, 1:2 or 1:3) in the MAPLE target, with the DPP-DTT layer being deposited at 25 k laser pulses.

The experimental parameters used in the MAPLE deposition (DPP-DTT:C60 weight ratio, the number of laser pulses) and the characteristics of the organic thin films, such as the thickness and roughness parameters (root mean square (RMS), roughness average (Ra)) inferred from the AFM investigations, are summarized in Table 1. It can be seen that the thickness of the organic thin films deposited by MAPLE are strongly dependent on the number of laser pulses. Thus, the values of the thickness are identical or very close in the case of the samples deposited at the same number of laser pulses (25 k or 90 k).

**Figure 2:**
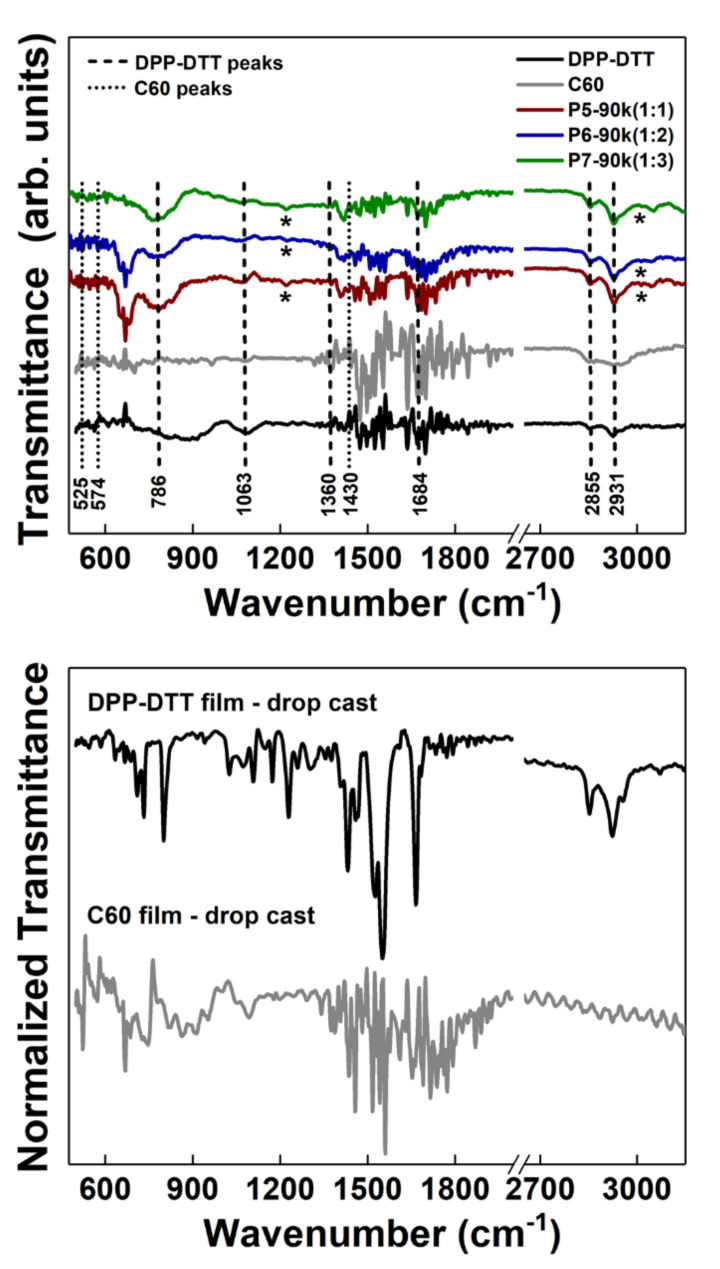
FTIR spectra of the thin films deposited by MAPLE on silicon and of the single components (DPP-DTT and C60) obtained by drop casting. * Peaks attributed to chloroform.

In order to evaluate the preservation or degradation of the chemical structures of both DPP-DTT and C60 during the laser processing, infrared spectroscopy was used for identifying their characteristic vibrations. Hence, Figure 2 presents the FTIR spectra of the thin films based on DPP-DTT and C60 as single components (at 25 k laser pulses) and as blends (at 90 k laser pulses) deposited by MAPLE. For comparison reasons, the FTIR spectra of the films based on the raw organic materials (DPP-DTT and C60) obtained by the drop cast technique are also shown. The specific vibration bands of each component are more easily observed in these layers due to their thickness. Usually, DPP-DTT in film form discloses a small number of its infrared bands [41]. Accordingly, in the FTIR spectra of the P5, P6, and P7 samples featured by a higher thickness, 190, 160, and 200 nm, respectively, the specific vibrations of this conjugated polymer can be identified at about 786 cm^−1^ associated with the C-H bonds (out of plane bending vibration) from the thiophene rings, 1063 cm^−1^ related to the C-N bonds (stretching vibration) from the DPP group, 1360 cm^−1^ attributed to the C-H bonds (in plane bending vibration) from the methyl units, 1684 cm^−1^ related to the C=O bonds (skeleton vibration), 2855 cm^−1^ correlated to the saturated C-H bonds (symmetrical stretching), and 2931 cm^−1^ linked to the C–H bonds (asymmetrical stretching) on the thiophene ring [42]. If the characteristic vibrations of the conjugated polymer are clearly identified in the FTIR spectra of the DPP-DTT:C60 thin films deposited by MAPLE, the typical vibrations of C60 located at about 525, 574, and 1430 cm^−1^ [43] can be barely noticed in these spectra due to their weak intensity.

Additionally, in the FTIR spectra of the investigated samples the appearance of the vibrations assigned to the chloroform molecules situated at about 1215 and 3019 cm^−1^ related to the C–H stretching and C–H bending, respectively [44], is due to the presence of traces of this solvent in the organic layers deposited by MAPLE. In a molecular dynamic simulation study regarding the ejection and transport of organic molecules in the MAPLE process, it was evidenced that some droplets on the substrate may not be completely free of solvent molecules, resulting in the deposition of organic films with solvent traces [45]. Based on the presence of the vibrational signatures of both organic compounds, DPP-DTT and C60, in the thin films deposited by MAPLE, it can be assumed that their chemical structure is preserved during the laser processing, no degradation process taking place.

**Figure 3:**
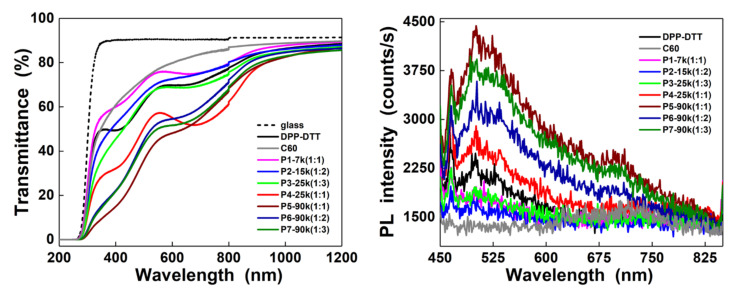
UV-VIS and PL spectra of the thin films deposited by MAPLE on glass and silicon, respectively.

Further, the optical properties of the thin films based on the DPP-DTT:C60 blends deposited by MAPLE were investigated by UV-VIS spectroscopy and photoluminescence. Thus, regardless of the weight ratio between the two organic components and the number of laser pulses involved in their MAPLE deposition, the UV-VIS spectra (Figure 3 left) and PL spectra (Figure 3 right) of the thin films based on DPP-DTT:C60 blends are dominated by the absorption attributed to the DPP-DTT in the visible range and by a broad weak emission band with a maximum at ~525 nm, respectively. The shape of the UV-VIS absorption spectrum of DPP-DTT, with two minima at ~420 and ~670 nm, is similar to that reported for the films based on this conjugated polymer with a low molecular weight deposited by spin-coating from chloroform [13,14,21,46]. Thus, the two absorption bands, one less intense between 360 and 500 nm and the other more intense between 500 and 1000 nm, are associated with the π-π* transitions and the ICT transition from the DTT unit to the DPP unit, respectively [47]. Additionally, in the UV-VIS spectrum of the DPP-DTT film deposited by MAPLE, a lower absorption can be noticed in comparison to the stronger absorption and sharper spectral features presented in the previous reports [13,14,25]. This outcome can be explained taking into account two aspects: the lower molecular weight of DPP-DTT, in agreement with the result reported in [25], and/or the lower concentration of the conjugated polymer (3% *w/v*) involved in the MAPLE process. Additionally, it can be mentioned that the increase in the weight ratio induces an attenuation effect in the two maxima absorption of the conjugated polymer. In the case of the other organic component, the UV-VIS spectrum of the C60 thin film deposited by MAPLE discloses a weak broad absorption band with a maximum at ~450 nm, specific to this compound [27].

Concerning the broad weak emission band from the visible range, this result is intrigued taking into account that DPP-DTT is not luminescent in this domain [48]. Yet, a similar emission was observed in the case of the MAPLE-deposited thin films based on poly(9,9-dioctylfluorene), another conjugated polymer, being related to the formation of some defects induced by the presence of solvent molecules in the organic layers during the laser process [49]. In our case, the appearance of this emission can be due to the chloroform traces from the MAPLE-deposited thin films based on DPP-DTT:C60 blends, their presence being evidenced by the FTIR measurements. Even the weak absorption between 400 and 500 nm linked to the presence of the fullerene cannot be clearly identified in the UV-VIS spectra of the thin films based on the blends being overlapped by DPP-DTT absorption; the PL spectra of the thin films deposited at 90 k laser pulses (the thicker layers) reveal a weak band centered at ~700 nm, this being the typical emission signature of C60 [50]. This emission band with a maximum at ~720 nm can be clearly observed in the PL spectrum of the C60 thin film, being specific to this compound [27]. Consequently, in the UV-VIS and PL spectra of the organic layers deposited by MAPLE can be identified the absorption bands associated with DPP-DTT and the emission bands attributed to C60, respectively.

**Figure 4:**
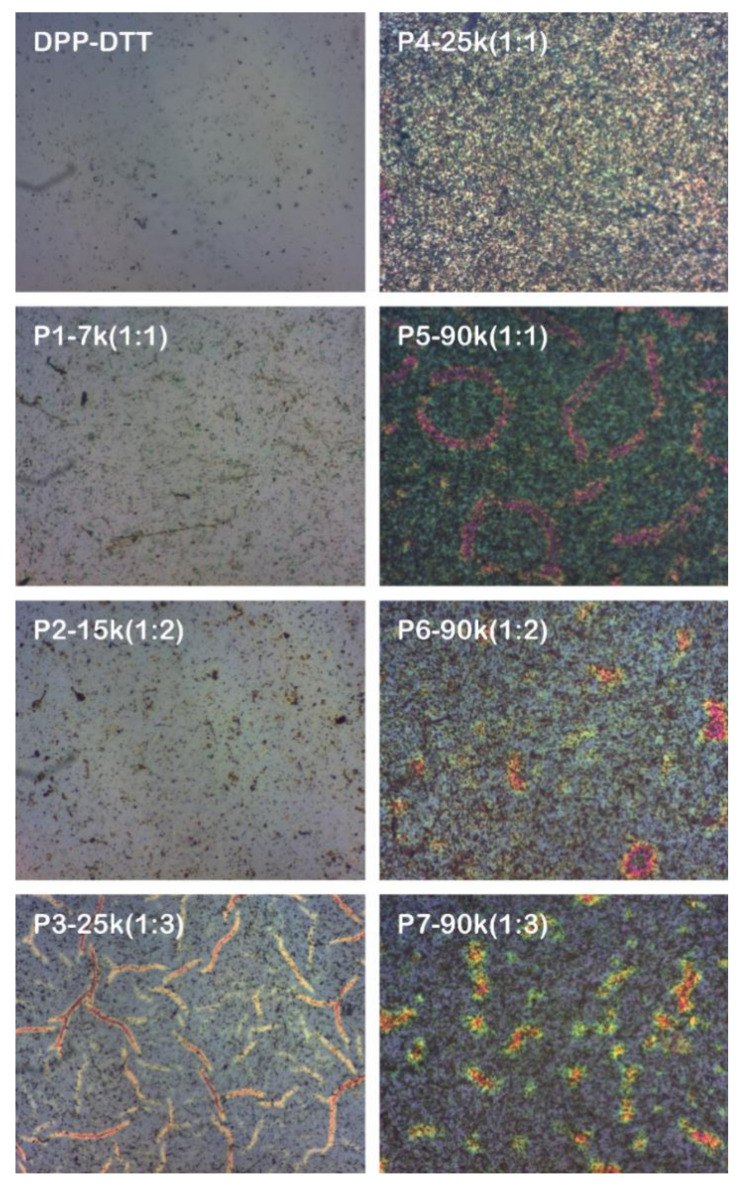
Optical images of the layers deposited by MAPLE on ITO/PEDOT:PSS (500×, 150 μm × 120 μm).

**Figure 5:**
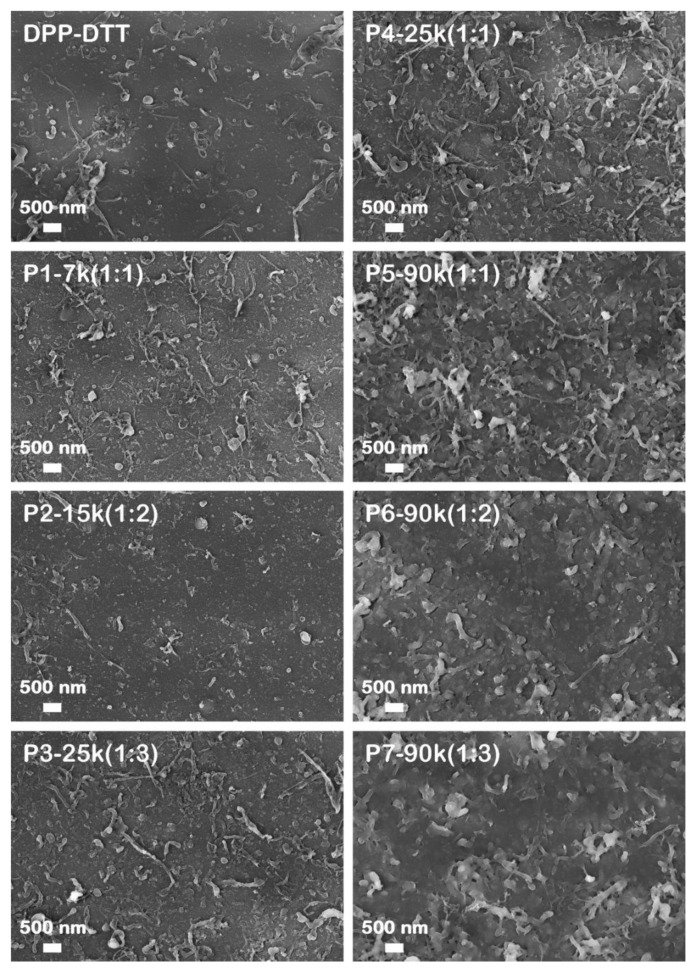
FESEM images of the layers deposited by MAPLE.

**Figure 6:**
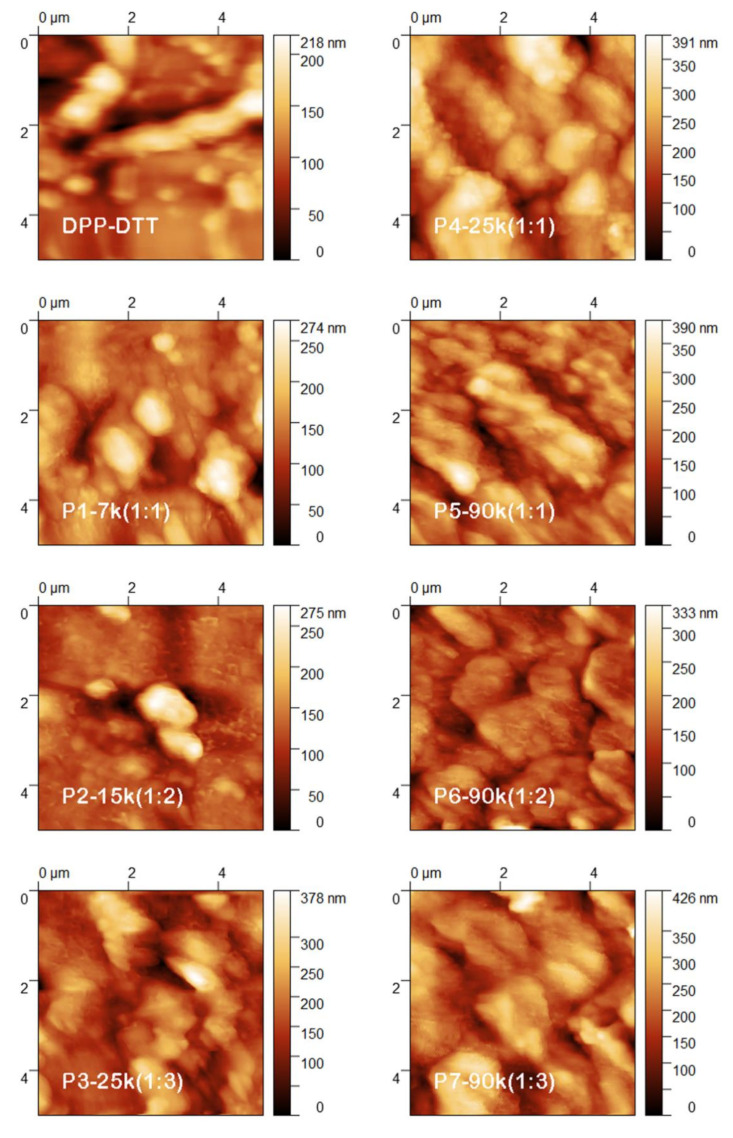
Topographic images of the layers deposited by MAPLE on ITO/PEDOT:PSS.

The layers based on the DPP-DTT and DPP-DTT:C60 blends deposited by MAPLE were also evaluated from a morphological point of view, with their optical, FESEM, and AFM images being presented in Figure 4, Figure 5, Figure 6, respectively.

For all investigated samples, the optical images (Figure 4) exhibit, on a large scale, an agglomeration tendency, this effect being augmented by the presence of C60 and by the increases in the number of laser pulses. Thus, the P3–P7 samples display large aggregated domains on the surface of the blend films.

The FESEM images (Figure 5) show that the surface of the organic films contains aggregates and fibril-like structures with different sizes.

Usually, in a MAPLE process, the aggregates originate from the mechanism of target ablation, this being responsible for the ejection of the polymer and solvent clusters toward the substrate while the fibril-like structures are observed in the films based on DPP-DTT obtained by spin-coating [51,52]. Additionally, some studies report on the ability of C60 molecules to form nanostructures (rods, wires, tubes, platelets, etc.) from the pristine C60 solution by a solution driven self-assembly process consisting in the solvent evaporation at room temperature, the morphology of these C60 nanostructures being tuned by using solvents in which C60 are more (toluene, dichlorobenzene) or less (carbon tetrachloride, chloroform) soluble [53,54,55]. Thus, the FESEM images of the DPP-DTT:C60 blend films evidence that the presence of fullerene favours the formation of a larger number of fibril-like structures in comparison with the layer based only on the conjugated polymer. Furthermore, in the DPP-DTT:C60 thin films deposited at a higher number of laser pulses it seems that an interpenetrating network architecture is formed. This result can be explained by the following possible mechanism: during the MAPLE deposition, the C60 molecules can form aggregates which through a self-assembly process can lead to the formation of fibril-like structures that further can form “bridges” between the DPP-DTT fibril structures which finally can lead to the formation of an interpenetrating network architecture in the thicker layers.

The AFM images (Figure 6) disclose also aggregates and fibril-like structures, in agreement with FESEM images. Further, the roughness parameters (RMS and Ra) were evaluated from the topographic images, their values being given in Table 1. In the following, the RMS values will be analysed in a comparative manner from both experimental parameters point of view: DPP-DTT:C60 weight ratio and number of laser pulses: i) for DPP-DTT (27 nm), P4 (64 nm) and P3 (53 nm) samples deposited at the same number of laser pulses (25 k), the addition of the fullerene results in a significantly increase in the RMS values, whereas the increase in the weight ratio from (1:1) to (1:3) leads to a low decrease in the RMS parameter; ii) for P1 (30 nm), P2 (35 nm) and P3 (53 nm) samples in which both experimental parameters were increased, an important increase in the RMS parameter is obtained for the layer deposited at high weight ratio (1:3) and high number of laser pulses (25 k); iii) for P1 (30 nm), P4 (64 nm) and P5 (54 nm) samples deposited at the same weight ratio (1:1) using different number of laser pulses, a notable increase in the RMS value is observed in the case of the layer obtained at 25 k while the increase at 90 k leads to a low decrease in the RMS parameter; iv) for P5 (54 nm), P6 (61 nm) and P7 (66 nm) samples deposited at the same number of laser pulses (90 k) using different weight ratio, the gradual increase in the weight ratio results in a gradual, but not substantial, increase in the RMS values.

Based on these data, it can be concluded that the presence of C60 in addition to DPP-DTT and the number of the laser pulses significantly influence the roughness parameters of the blend layers deposited by MAPLE. However, the increase in the DPP-DTT:C60 weight ratio does not affect in a major way the RMS values, due to the small size of the fullerene molecule in comparison with that of the DPP-DTT, the chains of the conjugated polymer favoring only a better arrangement of the fullerene molecules inside and at the surface of the film.

**Figure 7:**
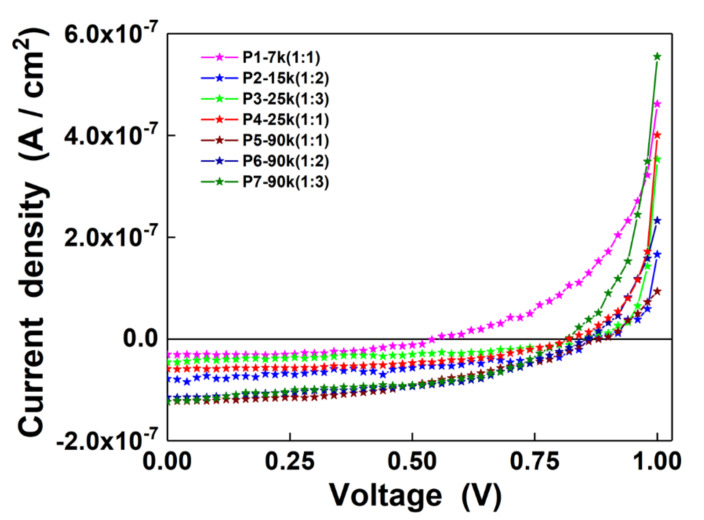
J-V characteristics of the structures developed on the thin films based on DPP-DTT:C60 blends deposited by MAPLE.

The J-V characteristics recorded under illumination of the structures developed on the DPP-DTT:C60 blend films obtained by MAPLE are presented in Figure 7. Additionally, the values of the electrical parameters, short-circuit current density (J_SC_), open circuit voltage (V_OC_), maximum power (P_max_), and fill factor (FF) corresponding to each investigated structures are given in Table 2.

Generally, in the polymeric solar cells, the V_OC_ value is linked to the offset between the HOMO level of the electron donor and the LUMO level of the electron acceptor [56]. Additionally, various studies report that the V_OC_ value can be influenced by the donor/acceptor interface area, light intensity, defect state, crystallinity, morphology, etc. [56,57]. In the case of the V_OC_ parameter, the structure based on P1 (the most thinner film) is featured by the lowest V_OC_, while, regardless of the DPP-DTT:C60 weight ratio and the thickness of the organic layers, a significant increase in the V_OC_ value was achieved for the other samples when the number of laser pulses increased. A similar result concerning the minimal influence of the thickness of the organic layers on the V_OC_ values is also evidenced for structures based on another conjugated polymer and fullerene-derivative compound, P3HT:PC71BM [58]. As regards the J_SC_ parameter, excepting the structure based on P2, a gradual increase in this electrical parameter was recorded for P1 to P7 samples. Interesting, for all structures based on thicker films (P5–P7 samples), the J_SC_ values are very close being higher than those of the structures involving the thinner films (P1–P4 samples). Several factors such as absorption properties, thickness layer, roughness can be responsible for this outcome. Thus, in the P3 film containing DPP-DTT:C60 in a 1:3 weight ratio, the small content of DPP-DTT and the thickness of the layer result in a weaker absorption which can be associated with a small number of excitons generated in the active layer. From the structures developed on P2 and P6 layers, deposited at different number of laser pulses but both containing DPP-DTT:C60 in 1:2 weight ratio, P2 sample exhibit the best electrical parameters (P_max_) from the thinner films, while the P6 sample presents the best device parameters from the all investigated samples. A similar outcome was observed in the case of thin films based on P3HT:C60 blends deposited by MAPLE using the same weight ratio [27]. Consequently, the addition of C60 can favor the formation of a larger interface between the conjugates polymer and fullerene. A higher increase in the C60 amount enlarges this interface and in the same time, also increases the layer thickness and its roughness affecting the charge carrier transport. Regarding the FF parameter, all investigated samples presents similar values ranging between 0.42 and 0.54, the highest value being obtained in the case of P1 sample (the most thinner film featured by a reduced roughness). The result can be explained taking into account that in the organic solar cell, the FF value is related to the mobility of the carriers, the thickness of the active layer and the morphology of the interfaces [59]. Previously studies shown that a high roughness and a low molecular weight of the DPP-DTT can decrease the electron and hole mobilities, leading to poor device performance, while a high concentration of the conjugated polymer featured also a high molecular weight results in films with adequate electrical properties [14,16,25]. Although the values of the electrical parameters of the developed structures were smaller in comparison with those of the films based on DPP-DTT and the fullerene derivative compounds deposited by spin-coating [14,25,26], they can be improved by modifying the MAPLE process parameters. Hence, by increasing the concentration of the conjugated polymer in the deposition target and by changing the number of the laser pulses, frequency, etc., the absorption properties of the active layers can be improved and their surface roughness can be reduced.

## 4. Conclusions

Thin films based on DPP-DTT and C60 blends were deposited by MAPLE technique, the influence of two experimental parameters (the weight ratio between the two components and the number of laser pulses used in the deposition process) on the properties of the obtained DPP-DTT:C60 blend layers being investigated. The presence of the vibrational fingerprints of both organic compounds, DPP-DTT and C60, in the organic layers deposited by MAPLE confirm that their chemical structure is preserved during the laser processing. Regardless the weight ratio between the two organic components and the number of laser pulses used during the laser-assisted deposition, in the UV-VIS spectra of the prepared DPP-DTT:C60 blend layers the absorption bands associated with DPP-DTT can be identified, whereas only in the PL spectra of the thicker blend films obtained at 90 k laser pulses can the emission bands attributed to C60 be observed. The FESEM images show that the surface of the organic films contains aggregates and fibril-like structures. Moreover, the aggregates seem to assemble into the fibril-like structure that further tends to form an interpenetrating network architecture in the thicker layers deposited at 90 k laser pulses. The roughness parameters of the obtained organic blend layers are significantly influenced by the presence of C60 in addition to DPP-DTT and the number of the laser pulses. However, the increase in the DPP-DTT:C60 weight ratio does not affect in a major way the RMS values due to the small size of the fullerene molecule in comparison with that of the DPP-DTT. Regardless the DPP-DTT:C60 weight ratio and the number of laser pulses, the current–voltage characteristics recorded, under illumination, of all the structures developed on the MAPLE-deposited films evidenced a photovoltaic cell behavior. The best electrical parameters were obtained for the sample deposited at 90 k laser pulses with DPP-DTT:C60 in a 1:2 weight ratio. This study proved the potential of the MAPLE technique to be used as a feasible approach in the deposition of the organic thin films based on conjugated polymers, with tailored properties which can be further integrated in organic photovoltaic devices.

## Figures and Tables

**Table 1:** Experimental parameters involved in the MAPLE deposition of the organic thin films and their characteristics.

Sample	Ratio	Number of the Laser Pulses	Thickness (nm)	RMS (nm)	Ra (nm)
DPP:DTT	-	25 k	35	27	21
P1	1:1	7 k	40	30	23
P2	1:2	15 k	40	35	24
P3	1:3	25 k	70	53	42
P4	1:1	25 k	70	64	52
P5	1:1	90 k	190	54	43
P6	1:2	90 k	160	61	46
P7	1:3	90 k	200	66	53

**Table 2:** Electrical parameters of the structures developed on the MAPLE-deposited layers.

Sample	V_OC_ (V)	J_SC_ (A/cm^2^)	P_max_ (W)	FF
P1	0.54	3.0 × 10^−8^	8.8 × 10^−9^	0.54
P2	0.86	7.8 × 10^−8^	2.9 × 10^−8^	0.43
P3	0.86	4.7 × 10^−8^	1.7 × 10^−8^	0.42
P4	0.82	5.9 × 10^−8^	2.5 × 10^−8^	0.52
P5	0.88	1.2 × 10^−7^	4.6 × 10^−8^	0.44
P6	0.86	1.1 × 10^−7^	5.0 × 10^−8^	0.53
P7	0.82	1.2 × 10^−7^	4.7 × 10^−8^	0.48
